# Scales to evaluate developmental stage and professional identity formation in medical students, residents, and experienced doctors

**DOI:** 10.1186/s12909-020-1942-y

**Published:** 2020-02-10

**Authors:** Masami Tagawa

**Affiliations:** Center for Innovation in Medical and Dental Education, Graduate School of Medical and Dental Sciences, Kagoshima University, 8-35-1 Sakuragaoka, Kagoshima, 890-8544 Japan

**Keywords:** Professional identity formation, Evaluation, Medical trainees, Socialization, Kegan’s model

## Abstract

**Background:**

To respond to the growing need to cultivate medical trainees with professional identity, it is necessary to evaluate professional identity formation (PIF) in medical trainees to understand their state of PIF and apply this to medical education. Previous qualitative studies indicated that Kegan’s human development model could explain medical trainees’ PIF. I proposed a development scale (DS) to quantitatively evaluate the degree of maturation and socialization as a physician; however, one scale is not enough to illustrate the helical and complex process of development.

**Methods:**

Using Kegan’s model as the conceptual framework, scales that evaluate stage 2, 3, and 4, and higher stage-specific attributes were developed using data collected in a self-administered questionnaire (322 respondents), reliability analysis, group comparison, and analysis of individual DS scores. The respondents were 4th- and 6th-year medical students and 2nd-year residents at Kagoshima University, and experienced medical doctors (instructors).

**Results:**

In addition to the DS, one self-administered questionnaire consisting of 27 items for stage 2, 3, 4, and higher stage-specific attribute scales was created. Students had the highest mean score in stage 2, and instructors had the highest mean score in stage 4 and higher stage scales. Individual analysis indicated that there were respondents with varied attributes in each group, that the average medical student might have inclusion preference typically seen at stage 3, and that the average instructor might have independent preference typically seen at stage 4 more than inclusion preference.

**Conclusions:**

Combining multiple stage attribute-specific scales and DS scores could quantify the complexity and divergent processes of PIF. These scales could provide meaningful information about individuals, groups, and education in terms of professional development that is different from assessment data of medical knowledge or professional skills.

## Background

As professional identity formation (PIF) of medical trainees is now a great concern to medical educators [1–6], evaluation of PIF, especially socialization and professional value formation, has become an important issue for medical education [7–15].

**Fig. 1:**
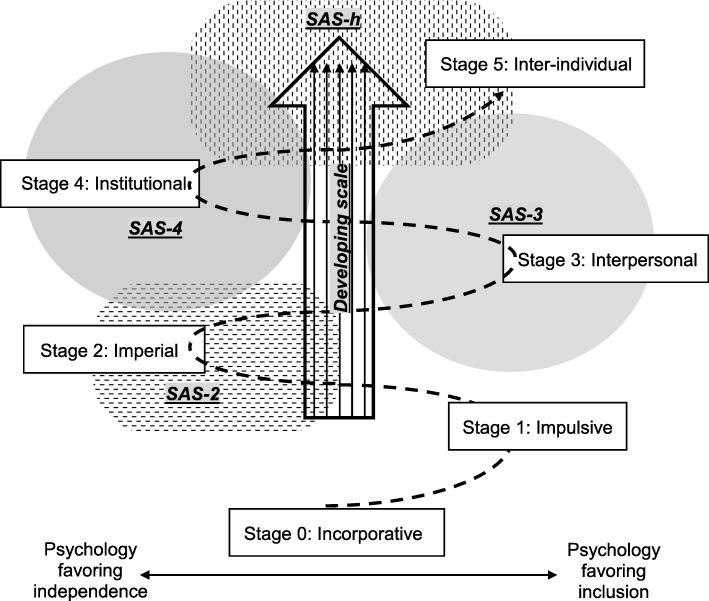
A modified Kegan’s helix of evolutionary truces (- - ▶) [reference 16], and expected scale direction and areas of evaluation. SAS: stage-specific attribute scale; SAS-2: stage 2-specific attribute scale; SAS-3: stage 3-specific attribute scale; SAS-4: stage 4-specific attribute scale; SAS-h: stage 4 and higher-specific attribute scale

Based on human development theories by Piaget, Kohlberg, Loevinger, Maslow, McCleland, Murray, Ericson and others, Kegan proposed a life-long developmental framework of the self into a moral and meaning-making entity [16]. Kegan illustrated changes in individual sense, perspectives, values, emotional control and reflection as part of the process of the development of relationships with others and society. Kegan’s model represents a 6-stage helical pathway of evolutionary truces that people follow in a psychological reciprocal process of favoring inclusion with and independence from others during their development (from stage 0 to 5) (Fig. 1).

Previous qualitative research indicated that Kegan’s model [16] can explain the development process in dentistry [1], the military [17, 18], and the legal profession [19], and theoretically, the model can also be applied to medical trainees [7, 13]. Previous qualitative research indicates that medical trainees are supposed to be in stages 2 to 4 [4, 7, 13]. Based on these studies, one scale (the Developing Scale, DS) to evaluate the overall degree of personal and professional development was developed [20]. This scale evaluates self-control as a professional, awareness of being a medical doctor, reflection as a medical doctor, execution of social responsibility, and external and internal self-harmonization.

Even though the DS could be a useful scale for evaluating PIF, one scale could not satisfactorily represent the helical and complex process of PIF, and attributes that characterize people at different developmental phases should be independently evaluated when determining PIF of individuals and target groups.

The purpose of this study was to develop scales to evaluate Kegan’s stage-specific attributes, and attempt to reveal medical trainees’ individual PIF, as well as group diversity.

## Method

To illustrate target individual and group PIF, four scales that cover the different stages of Kegan’s model were developed (Fig. 1). The following assumptions were used: evaluation of specific attributes for Kegan’s stage 2 to 5 would cover the entire process of PIF in medical trainees [4, 7, 13], and lower stage-specific attributes decrease and higher stage-specific attributes increase as the level of education and clinical experience advance.

Stage-specific attribute scales (SASs) were developed simultaneously along with the DS as follows: 1) an initial item pool with items that cover attributes from Kegan’s stage 2 to 5 (referencing previously reported manifestations of people at stage 2, 3, and 4 in the context of medical training and practice) was created; 2) a pilot questionnaire with essential and common items with medical context using items selected from the initial item pool was created; 3) the pilot questionnaire was administered to medical students, residents, and experienced medical doctors; 4) respondent data from the pilot questionnaire were used to elucidate item sets for proposed SASs using a reliability coefficient, and 5) means of proposed item sets from the different respondent groups were compared to confirm proposed SAS scores indicating development. Following this procedure, four SASs were developed.

### Initial item development

To create items for the four SASs, descriptions of medical trainees’ personal characteristics and behaviors or attitudes manifested in a professional context cited in previous studies [13, 17] were used.

To assess stage 2-specific attitudes and behaviors (stage 2-specific attribute scale: SAS-2), items describing an individual who took into account the views of others but whose own needs and interests predominate, whose norms were external rules, whose self-reflection was low, and whose emotions could overwhelm reason were used. To assess preference of inclusion typically seen at stage 3 (stage 3-specific attribute scale: SAS-3), items describing an individual who was able to view multiple perspectives simultaneously and subordinate self-interest and who was concerned about how others perceive him/her were used. To assess preference of independence typically seen at stage 4 (stage 4-specific attribute scale: SAS-4), items describing an individual who could assume a role and enter into relationships while assessing them in terms of self-authored principles and standards and who could define him/herself independently of others were used. To assess attributes expected at stage 4 or higher (stage 4 or higher-specific attribute scale: SAS-h), items describing an individual who clearly recognized professional roles; whose reason was in full control over needs, desires, and passion; who did not perceive him/herself as having a single identity and was open to other influences were used.

After creating and rewriting the items, 31 items to be used for the next round of data collection were selected. They consisted of 11 items for SAS-2, eight items for SAS-3, five items for SAS-4, and seven items for SAS-h. Of these SAS candidate items, 23 items for SAS-2, 4, and h were also used as DS candidates. Fifteen (items 1–15) satisfied the DS criteria and were used in the DS [20].

The questionnaire was self-administered and anonymous. Each item was scored on a 7-point Likert scale that ranged from 1 (completely inapplicable) to 7 (greatly applicable), and 4 was neutral. The questionnaire also asked about demographic characteristics (gender, age), as well as work experience and position for instructors.

### Data collection

From July 2016 to March 2018, the printed questionnaire was distributed by hand to 4th-year medical students about to start their clinical clerkship courses, 6th-year medical students who finished 1.5 years of all clinical clerkship courses, and residents in the last month of the 2-year residency program at Kagoshima University. The author did not have a direct relationship (i.e., instructor or supervisor) with any of the medical students and residents participating in this study at the time of data collection. The questionnaire was also distributed by mail to experienced medical doctors working in community hospitals or private clinics in Kagoshima Prefecture who engaged in undergraduate medical education as senior instructors. Questionnaires were anonymous and were returned by postal mail in January 2017 using the return envelope provided with the questionnaire.

### Data analysis for scale development

To develop each SAS, the reliability (Cronbach’s alpha) of candidate items was analyzed and reliable item sets were explored.

After item sets for all scales were fixed, confirmation of whether the lower stage-specific and higher stage-specific attribute scales could differentiate between different developing groups was performed using the average scores of the SASs in the four respondent groups. Furthermore, medical trainees’ PIF that SASs could provide, such as the stage of the respondent groups and diversity among groups was analyzed.

SPSS version 23 (IBM, New York, NY) was used for all data analyses.

## Results

### Demographic characteristics of the respondents

The same data were used as for the DS development [20]. Prior to the analysis, 14 respondents who chose option 4 (neutral) as the response for 27 items or more (87%) or for 23 sequential items (74%) were excluded as invalid data. Data for a total of 322 respondents (response rate 53.7%), including 118 (response rate 47.8%) 4th-year medical students and 120 (response rate 51.5%) 6th-year medical students at Kagoshima University School of Medicine, 47 (response rate 73.4%) 2nd-year residents at Kagoshima University Hospital, and 37 (response rate 66.1%) medical doctors at community hospitals and private clinics who served as instructors for medical students were included in this research. The mean ages of 4th-year medical students, 6th-year medical students, residents, and instructors were 24.2, 25.4, 29.7, and 55.2 years, respectively. The mean length of clinical experience among instructors was 29.3 years (standard deviation 6.2 years, range 15–40 years).

### Development of SASs

Using the items related to key attributes of each stage, item sets with the highest Cronbach’s alpha for SASs were explored. Cronbach’s alpha for the proposed SAS-2, SAS-3, SAS-4, and SAS-h were 0.66 (11 items), 0.53 (six items), 0.61 (three items), and 0.63 (six items), respectively (Table 1, Additional file 1). Items 1 to 14 were identical to the DS items, and the coding direction differed depending on the scales.

**Table 1:** Items used for the four SASs, and mean item scores for each respondent group

		Items used for scales				Item score								
							4th-year medical students		6th-year medical students		2nd-year residents		Instructors	
Item		Stage 2	Stage 3 Inclusion	Stage 4 Independence	Higher		Mean	(SD)	Mean	(SD)	Mean	(SD)	Mean	(SD)
1	I cannot tolerate that colleagues who sympathize with my actions have a different mindset from me.	F				R	5.56	(1.244)	5.51	(1.270)	5.51	(1.502)	**5.65**	(1.086)
2	I find it difficult to suppress my desires and act rationally.	F				R	5.05	(1.437)	5.08	(1.406)	**5.45**	(1.442)	5.41	(1.322)
3	It is difficult for me to adjust and act according to the different values of each medical professional and the demands for physicians.	F			R	R	4.19	(1.377)	4.20	(1.268)	4.34	(1.550)	**5.00**	(1.202)
4	I have never thought about the reasons or principles behind the required code of conduct.	F				R	4.56	(1.251)	4.33	(1.238)	4.51	(1.249)	**4.86**	(1.316)
5	I am sometimes unable to do something I was not interested in despite understanding its necessity.	F				R	3.84	(1.313)	4.23	(1.454)	**4.43**	(1.678)	4.27	(1.347)
7	I behave correctly as a physician on a daily basis.				F	F	4.25	(0.999)	4.24	(1.108)	5.00	(1.319)	**5.41**	(1.235)
8	I am aware of my position as a physician.				F	F	4.97	(1.330)	4.68	(1.361)	6.26	(1.113)	**6.65**	(0.633)
9	I have accepted the words of gratitude and the frustration and anger of patients as a personal evaluation of myself.		F			F	4.86	(1.080)	5.18	(1.092)	5.13	(0.992)	**5.57**	(0.835)
10	I consider long-term significance and concerns when I think about what I should do.				F	F	5.29	(1.087)	5.18	(1.112)	5.28	(1.136)	**5.54**	(0.767)
11	I have used my own beliefs and ideals as a standard to evaluate my own actions as a physician.			F		F	4.58	(1.024)	4.62	(1.030)	4.43	(1.156)	**4.89**	(0.994)
12	If I were able to play a role in improving society and organizations, I would be satisfied even if I did not receive individual recognition.				F	F	4.23	(1.386)	4.04	(1.411)	4.28	(1.347)	**4.57**	(1.425)
13	I induce action in the people around me based on the principles I believe in to fulfill my role as a physician.			F		F	4.48	(1.027)	4.38	(1.117)	4.43	(1.078)	**5.11**	(0.843)
14	I take on various roles in accordance with the demands of society.				F	F	4.57	(1.199)	4.28	(1.159)	5.13	(0.947)	**5.49**	(0.901)
16	Adhering to the rules and code of conduct of one’s institution is the basis of correct behavior as a physician.	F				R	3.08	(1.255)	**3.29**	(1.205)	3.26	(1.224)	3.27	(1.465)
17	I recall and confirm the systems and rules for acting appropriately as a physician.	F				R	2.85	(0.993)	3.05	(1.052)	**3.09**	(1.299)	3.08	(1.278)
18	The colleagues I can be close friends with are those who have the same concerns and interests as me.	F				R	3.42	(1.555)	3.26	(1.411)	**3.62**	(1.468)	3.49	(1.557)
19	The way senior physicians act towards me is more important than how they feel about me.	F				R	**3.93**	(1.153)	**3.93**	(1.047)	3.85	(1.302)	3.73	(1.521)
20	Good test results and qualifications are important evaluation of self.	F				R	4.28	(1.377)	3.85	(1.400)	**4.70**	(1.600)	4.54	(1.445)
21	It is important to me that specific actions required of me in each medical setting are clearly specified.	F				R	3.21	(0.968)	3.47	(1.141)	3.46	(1.312)	**3.86**	(1.206)
22	I act with respect for the opinions of my colleagues when I have a conflict of opinion with those around me.		F			R	3.37	(0.985)	3.39	(0.938)	3.49	(1.249)	**3.51**	(0.731)
23	I take great care not to offend senior physicians or colleagues.		F			R	3.16	(1.147)	2.98	(0.970)	2.89	(1.127)	**3.46**	(1.238)
24	I first consider how to share responsibilities with colleagues in order to fulfill duties required of me.		F			R	3.21	(1.146)	**3.43**	(1.051)	3.17	(1.028)	3.08	(1.211)
25	I have set aside my own interests to meet the ideals and expectations of society.		F			F	3.87	(1.121)	3.85	(1.042)	3.98	(1.482)	**4.41**	(1.117)
26	When I am acknowledged by senior physicians or colleagues, I feel that I am being recognized.		F			R	2.77	(1.086)	2.73	(1.115)	**3.17**	(1.324)	3.11	(1.329)
27	I am working to improve my behavior as a physician by constantly analyzing and reflecting on my own actions.			F		F	4.78	(0.984)	4.83	(1.064)	4.89	(1.068)	**5.03**	(0.986)
Number of respondents		317	319	321	319	118 (117)			120 (119)		47 (46)		37	
Number of items		11	6	3	6									
Cronbach’s α		0.66	0.53	0.61	0.63									

*SAS* stage-specific attribute scale, *SD* standard deviation

Direction of coding: *F* forward, *R* reverse

Bold indicates the highest score among the respondent groups

### Confirmation of SASs by comparing respondent groups

Table 2 shows average scores of proposed SASs as well as total DS scores of the 15 items for each respondent group.

**Table 2:** Mean scores of DS and the four SASs for each respondent group

	4th-year medical students			6th-year medical students			2nd-year residents			Instructors		
	*N*	Mean	(SD)	*N*	Mean	(SD)	N	Mean	(SD)	*N*	Mean	(SD)
DS	115	68.1	(8.12)	119	67.6	(6.96)	47	72.2	(8.78)	37	78.1	(9.24)
SASs												
SAS-2	117	4.01	(0.60)	118	3.99	(0.57)	45	3.82	(0.76)	37	3.71	(0.68)
SAS-3	115	4.71	(0.56)	120	4.75	(0.57)	47	4.73	(0.76)	37	4.80	(0.62)
SAS-4	117	4.63	(0.80)	120	4.61	(0.74)	47	4.58	(0.84)	37	5.01	(0.70)
SAS-h	116	4.60	(0.76)	119	4.43	(0.59)	47	5.05	(0.77)	37	5.44	(0.60)

*DS* developing scale, *N* number of respondents, *SD* standard deviation, *SAS* stage-specific attribute scale, *SAS-2* stage 2-specific attribute scale, *SAS-3* stage 3-specific attribute scale, *SAS-4* stage 4-specific attribute scale, *SAS-h* stage 4 or higher-specific attribute scale

Medical students’ SAS-2 mean scores were higher than those of residents and instructors, and instructors’ SAS-4 and SAS-h mean scores were higher than those of students and residents. Instructors had increasing scores from the lowest stage (SAS-2) to highest stage (SAS-h).

Univariate analysis of variance of each SAS score indicated that respondent group was a significant variable for SAS-2 score and SAS-h score (SAS-2 score, *p* = 0.03; SAS-3 score, *p* = 0.70; SAS-4 score, *p* = 0.37; SAS-h score, *p* < 0.01) whereas gender was not a significant variable in any of the four SASs (SAS-2 score, *p* = 0.10; SAS-3 score, *p* = 0.07; SAS-4 score, *p* = 0.33; SAS-h score, *p* = 0.87).

### Confirmation of SASs using DS score

Since the SASs and DS utilized the same items, SAS-2 scores should negatively correlate and SAS-h scores should positively correlate with DS scores.

**Fig. 2:**
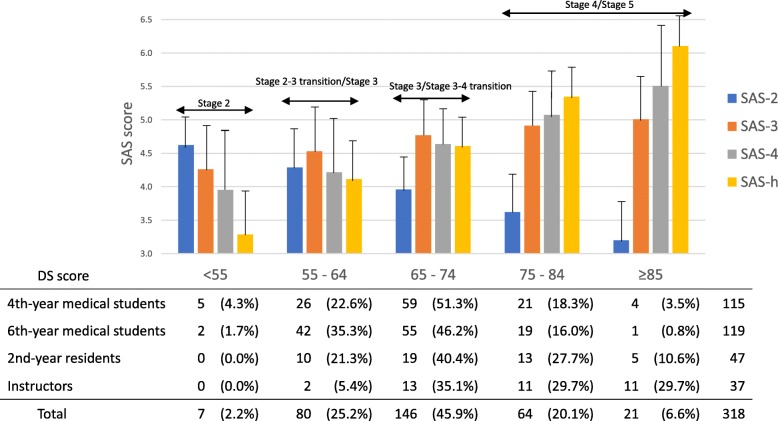
Mean and standard deviation of SAS scores in five DS score classifications and number of respondents DS: developing scale; SAS: stage-specific attribute scale; SAS-2: stage 2-specific attribute scale; SAS-3: stage 3-specific attribute scale; SAS-4: stage 4-specific attribute scale; SAS-h: stage 4 and higher-specific attribute scale

To analyze whether all four SAS scores were related to the DS, which evaluates overall maturation and socialization related to PIF, as theoretically expected, I examined SAS scores in the five DS score classifications (Fig. 2). As for DS scores of 54 or less (*n* = 7), SAS-2 mean score was the highest among the four SASs, whereas SAS-4 and SAS-h were both lower than 4 (i.e. neutral). As for DS mean scores of 85 or more (*n* = 21), SAS-4 and SAS-h were higher than 5.5, and SAS-h was the highest and SAS-2 was the lowest among the four SASs. As DS score increased from 55 to 84, SAS score transitioned from a pattern of high SAS-3 mean score to high SAS-4 and SAS-h mean scores and low SAS-2 mean scores.

### Comparison of individual SAS scores

**Fig. 3:**
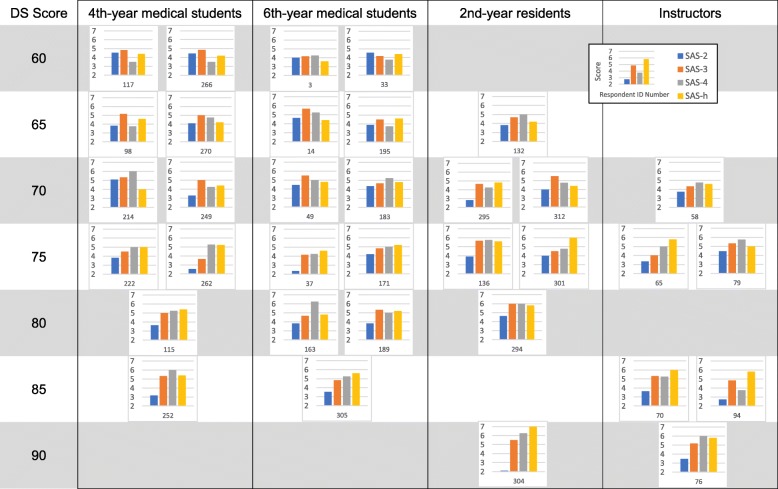
SAS scores of respondents with DS scores of 60, 65, 70, 75, 80, 85, and 90. If there were three or more respondents with these DS scores in each respondent group, two respondents from each group were randomly selected DS: developing scale; SAS: stage-specific attribute scale; SAS-2: stage 2-specific attribute scale; SAS-3: stage 3-specific attribute scale; SAS-4: stage 4-specific attribute scale; SAS-h: stage 4 and higher-specific attribute scale

To clarify the scale function at the individual level and the appropriateness of applying the scales to groups, I analyzed individual scores for SASs with the same DS scores. Figure 3 shows the four SAS scores of respondents whose DS scores were 60, 65, 70, 75, 80, 85, or 90. If there were several respondents with these DS scores in each respondent group, two respondents from each group were randomly selected.

For example, among a total of 16 respondents with a DS score of 70 (four 4th-year medical students, eight 6th-year medical students, three residents, and one instructor), seven respondents’ highest score was in SAS-3 (e.g. ID = 249, 49, and 312), three respondents’ highest score was in SAS-4 (e.g. ID = 214, 183, and 58) and three respondents’ highest score was in SAS-h (e.g. ID = 295) (Fig. 3).

Actually, the scores and patterns of SASs varied among individuals with the same DS score among respondents in the same group. Independent from DS score, some respondents showed high SAS-3 and SAS-h scores with low SAS-4 scores (inclusion pattern; e.g. ID = 117, 266, 98, 195, and 94), while others showed high SAS-4 scores with low SAS-3 and SAS-h scores (independent pattern; e.g. ID = 214, 163, and 252).

There were also tendencies indicated by the group mean scores. Many respondents with DS scores of 60 or 65 had a highest SAS-3 score among the four SASs, and most respondents with a DS score of 75 or higher had a highest SAS-4 or SAS-h score among the four SASs.

## Discussion

Previous research [7, 13] indicated that Kegan’s model could explain individual medical trainees’ personal and professional development. One advantage of using this model as a conceptual framework for scale development is its applicability to individuals in any program and specialty, or in any position, because it describes general lifelong human development in relationships with others and society [16]. However, because a helical and reciprocal process was proposed, one scale score might not adequately represent the complex and divergent pathway of professional development and could not provide meaningful insight into the PIF of an individual or group. To respond to this issue, I propose four SASs, in addition to the DS, provided in one self-administrated questionnaire with a total of 27 items.

Comparing the respondent groups revealed that SAS-2 score decreased and SAS-h score increased as training advanced from students to residents and even more to instructors. This result suggested that SAS-2 and SAS-h might be able to differentiate respondents at low or high stages even though the reliability coefficients were not high.

On the other hand, stage 3-specific inclusion preferences, such as “rely on others” and stage 4-specific independence preference, such as “behave according to own values”, were opposite attributes. As a result, group mean scores of SAS-3 and SAS-4 might be reflected by the ratio of constituent members at stage 3 and stage 4, influenced by a few respondents with extreme attributes, and indicative of neutral characteristics. In fact, individual score analysis indicated there were respondents suspected to have different attributes as well as individuals at different stages within the same respondent group. Even though this limitation existed in the group comparison, the mean scores of SAS-3 and SAS-4 in each group suggested that students might have inclusion preference typically seen at stage 3 more than independence, and instructors might have independent preference typically seen at stage 4 more than inclusion preference.

Basically, individual values and attitudes are complex and there are transition phases between stages. Each SAS did not correspond exactly to Kegan’s stages, but did indicate the tendency of key attributes related to staging. Information provided by the SASs is informative for discussing PIF, and illustrates that the four SASs are valid scales for this purpose.

People with low DS scores might be expected to be at a lower stage, and people with high DS scores should be at higher stages. As shown in Fig. 2, respondents with DS scores of less than 55 were expected to be at stage 2. Respondents with DS scores of 75 or higher were expected to be at stage 4 or 5.

The results of this research indicated that the average medical student in this study was at stage 3 and they ranged from stage 2 to 4. The average instructor in this study was at stage 4 or higher, and few instructors were at stage 2. These results were compatible with theoretical hypotheses on medical trainees [7, 13], and a qualitative interview analysis of law and dental students (from stage 2 to stage 4) [19].

Even though each of the scales developed in this study requires greater validity and higher reliability, the combination of scale scores could be an indicator of PIF that knowledge and skill assessment or behavior observation cannot provide. Analysis of each respondent’s scale scores, which represent individual attitudes and values and actual behaviors, should be investigated in the future.

## Limitations

The SASs had low reliability because of the limited number of items under the practical restrictions of the questionnaire compared to the complex and divergent process of PIF. I could not conclude whether the broad score range and differences among the four SAS scores were due to characteristics of the respondents’ PIF or error in an unreliable scale.

All items were written in Japanese and all respondents were located in Kagoshima, Japan. Long-term prospective studies and research in other locations are required to confirm scale sensitivity and improve usability.

## Conclusions

Multiple scales evaluating different developmental stage-specific attributes, combined with one scale evaluating degree of maturation and socialization, might provide meaningful information about individual and group PIF. Young medical trainees, such as medical students and residents, were in the process of PIF.

## Supplementary information

**Additional file 1:** The Questionnaire of professional identity formation, including DS, SAS-2, SAS-3, SAS-4, and SAS-h

## Data Availability

All data are stored according to agreements with the participants and ethical standards and are available upon reasonable request to the author.

## Abbreviations

DS: Developing scale
PIF: Professional identity formation
SAS: Stage-specific attribute scale

## References

[1] Bebeau MJ. Evidence based character development. In: Kenny NP, Shelton WN, eds. Lost virtue professional character development in medical education. Oxford, England: Elsevier; 2006.

[2] Cooke M, Irby DM, O’Brien BC. Educating physicians: a call for reform of medical school and residency. San Francisco, Calif: Jossey-Bass; 2010.

[3] Bleakley Alan, Bligh John, Browne Julie (2011). New Forms of Identity in a Runaway World of Medicine. Medical Education for the Future.

[4] Cruess RL, Cruess SR, Boundreau JD, Snell L, Steinert Y (2014). Reframing medical education to support professional identity formation. Acad Med.

[5] Wilson I, Cowin LS, Johnson M, Young H (2013). Professional identity in medical students: pedagogical challenges to medical education. Teach Learn Med.

[6] Irby DM, Cooke M, O’Brien BC (2010). Call for reform of medical education by the Carnegie foundation for the advancement of teaching: 1910 and 2010. Acad Med.

[7] Javis-Selinger S, Pratt DD, Regehr G (2012). Competency is not enough: integrating identity formation into the medical education discourse. Acad Med.

[8] Pitkala KH, Mantyranta T (2003). Professional socialization revised: medical students’ own conceptions related to adoption of the future physician’s role—a qualitative study. Med Teacher.

[9] Pratt MG, Rockmann KW, Kaufmann JB (2006). Constructing professional identity: the role of work and identity learning cycles in the customization of identity among medical residents. Acad Manag J.

[10] Hafferty FW. Professionalism and the socialization of medical students. In: Cruss RL, Cruess SR, Steinert Y, eds. Teaching medical professionalism. New York, NY: Cambridge University Press; 2009.

[11] Weaver R, Peters K, Koch J, Wilson I (2011). ‘Part of the team’: professional identity and social exclusivity in medical students. Med Educ.

[12] Frost HD, Regehr G (2013). “I am a doctor”: negotiating the discourses of standardization and diversity in professional identity construction. Acad Med.

[13] Cruess RL, Cruess SR, Boundreau JD, Snell L, Steinert Y (2015). A schematic representation of the professional identity formation and socialization of medical students and residents: a guide for medical educators. Acad Med.

[14] Holden MD, Buck E, Luk J, Ambriz F, Boisaubin EV, Clark MA, Mihalic AP, Sadler JZ, Sapire KJ, Spike JP, Vince A, Dalrymple JL (2015). Professional identity formation: creating a longitudinal framework through TIME (transformation in medical education). Acad Med.

[15] Sharpless J, Baldwin N, Cook R, Kofman A, Morley-Fletcher A, Slotkin R, Wald HS (2015). The becoming: students’ reflections on the process of professional identity formation in medical education. Acad Med.

[16] Kegan R. The evolving self: problem and process in human development. Cambridge, Mass: Harvard University Press;1982.

[17] Forsythe GB (2005). Identity development in professional education. Acad Med.

[18] Bartone PT, Snock SA, Forsythe GB, Lewis P, Bullis RC (2007). Psychosocial development and leader performance of military officer cadets. Leadership Q.

[19] Monson VE, Hamilton NW (2011). Entering law students’ conceptions of an ethical professional identity and the role of the lawyer in society. J Legal Prof.

[20] Tagawa M (2019). Development of a scale to evaluate medical professional identity formation. BMC Med Educ.

